# The Association between Obesity and Reduced Weight-Adjusted Bone Mineral Content in Older Adults: A New Paradigm That Contrasts with the Obesity Paradox

**DOI:** 10.3390/nu16030352

**Published:** 2024-01-25

**Authors:** Antonino De Lorenzo, Massimo Pellegrini, Paola Gualtieri, Leila Itani, Giulia Frank, Marwan El Ghoch, Laura Di Renzo

**Affiliations:** 1 Section of Clinical Nutrition and Nutrigenomic, Department of Biomedicine and Prevention, University of Tor Vergata, Via Montpellier 1, 00133 Rome, Italy; delorenzo@uniroma2.it (A.D.L.); laura.di.renzo@uniroma2.it (L.D.R.); 2 Centre for the Study of Metabolism, Body Composition, and Lifestyle, Department of Biomedical, Metabolic and Neural Sciences, University of Modena and Reggio Emilia, 41125 Modena, Italy; massimop@unimore.it; 3 Department of Nutrition and Dietetics, Faculty of Health Sciences, Beirut Arab University, Riad El Solh, Beirut 11072809, Lebanon; l.itani@bau.edu.lb; 4 PhD School of Applied Medical-Surgical Sciences, University of Tor Vergata, Via Montpellier 1, 00133 Rome, Italy; giulia.frank@ymail.com

**Keywords:** obesity, bone mineral content, older adults, trunk fat, appendicular lean mass

## Abstract

The relationship between body weight and bone mass in the elderly remains unclear, and whether obesity is a protective factor is still a matter of debate. For this reason, the aim of this study is to assess the association between body mass index (BMI) and bone mineral content adjusted by body weight, expressed as a percentage (w-BMC%), and to test the validity of the obesity paradox in this context. A cohort of 1404 older adults was categorized according to the World Health Organization’s BMI cut-off points and completed a total and segmental body composition measurement by means of a dual X-ray absorptiometry scan. Individuals with obesity displayed a lower mean w-BMC% (3.06 ± 0.44%; 2.60 ± 0.37%) compared to those who were normal-weight (3.95 ± 0.54%; 3.38 ± 0.48%) and overweight (3.06 ± 0.44%; 3.04 ± 0.37%) in both genders. Linear regression analysis also showed a negative association between BMI and w-BMC% in males (β = −0.09; *p* < 0.001) and females (β = −0.06; *p* < 0.001). Finally, among individuals with obesity, and after adjusting for age, the linear regression models revealed a significant decrease of 0.75% and 0.28% in w-BMC% for every one-unit increase in the trunk fat/appendicular lean mass ratio in both males (β = −0.749; *p* < 0.0001) and females (β = −0.281; *p* < 0.001). In conclusion, we suggest a new paradigm regarding the impact of obesity on bone mass, in which the former does not appear to be a protective factor of the latter, especially in individuals with central obesity and low muscle mass.

## 1. Introduction

Obesity is a chronic disease that is widespread in many countries, especially those with a Western lifestyle [1]. This prompted the World Health Organization (WHO) to introduce the term “globesity”, combining “global” with “obesity”, to emphasize the extent to which this problem has become relevant worldwide [2,3]. In fact, obesity is considered a major risk factor for several medical (i.e., type 2 diabetes, dyslipidemia, and hypertension) [4] and psychosocial (i.e., depression, anxiety, and eating disorders, as well as an impaired health-related quality of life (HRQoL)) morbidities [5,6], which may unavoidably lead to increased mortality rates [7]. For this reason, and despite their frequent lack of success [8], obesity guidelines recommend a wide range of weight loss interventions [9,10], including recently developed anti-obesity drugs [11]. Osteoporosis and fracture risk in older people pose another challenging health problem, with global estimates that 50% of females and 20% of males aged over 50 years will experience a fracture related to reduced bone mass, resulting in a significant total economic burden [12,13].

Taking into consideration both conditions (i.e., obesity and osteoporosis) [14], the impact of body weight on bone health is still unclear, since the data provided in the literature are controversial [15]. Some studies have reported a positive association between body mass index (BMI) and bone mass, in which obesity was found to be associated with increased bone mineral density (BMD) [16], suggesting that the former (i.e., obesity) acts as a protective factor against the risk of fracture [17] and supporting the paradigm of the “obesity paradox” [18,19], especially in older adults [20]. On the other hand, other scientific evidence has shown a greater risk of fractures in patients with obesity [21,22]. This introduced a new paradigm among researchers that contrasts with the previous one (i.e., the obesity paradox), which describes a complex relationship between obesity and bone health, hypothesizing that patients with obesity, especially those with central fat distribution and a lower muscle mass, appear to have an impaired bone status (i.e., low bone mass, higher risk of fracture, etc.) rather than a protected one [23].

The reason behind the discrepancy between the findings is still unclear. However, recent reports have claimed that the use of traditional diagnostic criteria for osteoporosis, based only on areal BMD, may lead to failure to diagnose osteoporosis in individuals with large bones (i.e., obesity) [24], as their high BMD values do not necessarily imply that they have stronger bones with a lower risk of fracture [25]. Some studies suggested a new adjusted index of bone mineral content (BMC) to make up for the shortcomings of BMD in osteoporosis diagnosis. One of the main variables to adjust was body weight, which was found to be a key determinant of bone mass [26,27]. For this reason, the standardization of BMC, taking into account body weight or BMI, should be investigated [24,28].

Based on these considerations, the aim of this study is (i) to assess bone mass across the BMI categories in older adults of both genders, taking into account the standardized BMC after adjustment by body weight, expressed in percentage (w-BMC%), as suggested elsewhere to avoid any interpretation bias across the BMI categories [24], and (ii) to clarify if obesity has any protective role in bone health and therefore to confirm or disprove the concept of the “obesity paradox”. We hypothesize that obesity is not a protective factor for bone mass but is associated with lower weight-adjusted BMC, especially in those individuals with central obesity and low muscle mass.

## 2. Materials and Methods

### 2.1. Participants and Design of the Study

This research involved a retrospective investigation that took the form of a single-center study. Individuals were pooled from an initial large cohort in which patients were consecutively and voluntarily recruited in the Nutritional Unit of the Department of Biomedicine and Prevention situated at the University of Rome “Tor Vergata” in Italy during the period from June 2018 to May 2022. The inclusion criteria were (i) having an age > 60 years, (ii) being normal-weight, overweight, or with obesity, according to the WHO BMI cut-off points (i.e., ≥18.5–24.9 kg/m^2^, ≥25–29.9 kg/m^2^, ≥30.0 kg/m^2^, respectively) [29], and (iii) completing a body composition test by means of dual X-ray absorptiometry (DXA). Patients were excluded if they were (i) aged <60 years or (ii) underweight, according to the WHO BMI cut-off points (i.e., <18.5 kg/m^2^), pregnant or lactating, taking medication that affects body weight or composition, suffering from severe psychiatric disorders, or presenting with medical comorbidities associated with weight loss (e.g., cancers). Accordingly, a total of *n* = 1404 individuals were included, and the post hoc power analysis using G*Power (version 3.1.9.2) for this sample gave a power of 1 for regression analysis in males or females from all BMI categories and a power of 0.9 for those with obesity [30].

Approval from the ethics committee was obtained from the Calabria Region Center Area Section (Register Protocol No. 97, 20 April 2023). All the patients’ data were treated according to European/Italian privacy laws and informed written consent was obtained.

### 2.2. Body Weight and Height

Body weight and height were measured by a medical doctor involved in the study with individuals fasting before breakfast, wearing light clothes and no shoes, using an electronic weighing scale (SECA 2730-ASTRA, Hamburg, Germany) and a stadiometer. The BMI was then calculated according to the standard formula of body weight measured in kilograms, divided by the square of the height in meters [29]:

BMI (kg/m^2^) = Body weight (kg) ÷ height^2^ (m)

### 2.3. Body Composition

Body composition was determined using a DXA (DXA, GE Medical Systems) fan beam scanner, which assessed both the whole and regional compartments (arms, trunk, and legs) in terms of fat, lean, and bone mass. Standard DXA quality control and calibration measures were performed prior to each testing session. Individuals were asked to abstain from any form of physical activity in the 24 h prior to the measurement, and before the test to remove all clothing except for undergarments including shoes, socks, and metal items prior to being positioned on the DXA table. Scans were performed with the individuals in a supine position. The entire body was scanned beginning from the top of the head and moving in a rectilinear pattern down the body to the feet, and the effective radiation dose from this procedure was approximately 0.01 millisieverts (mSv). The mean measurement time was 15 min and the average was 20 min. In this study, we considered the following variables:

(a) Body fat (BF) = total body fat expressed in kg;
(b) BF% (BF as a percentage of the total mass) = (BF ÷ body weight) ∗ 100;
(c) Trunk fat = total trunk fat expressed in kg;
(d) Trunk fat% = (trunk fat ÷ BF) ∗ 100;
(e) Lean mass (LM) = total lean mass, bone excluded, expressed in kg;
(f) LM% (LM as a percentage of the total mass) = (LM ÷ body weight) ∗ 100;
(g) Appendicular lean mass (ALM) = total lean in arms and legs with bone excluded, expressed in kg);
(h) Total bone mineral content (BMC) = total amount of minerals in bone expressed in kg;
(i) w-BMC% (BMC adjusted by body weight expressed as a percentage) = (BMC ÷ body weight) ∗ 100. As BMC varies with weight, BMC% serves as a standardized index accounting for between-subject weight variability [24];
(j) Trunk fat/appendicular lean mass ratio: trunk fat ÷ appendicular lean mass, to create a combined variable that expresses the central fat distribution and muscle mass in the extremities.

### 2.4. Statistical Analysis

The results are presented as means and standard deviations for continuous variables and frequencies and proportions for categorical ones. Student’s *t*-test and an analysis of variance (ANOVA) were performed to calculate the mean comparison between the different BMI categories and males and females, respectively. The chi-squared test for independence was utilized to explore the distribution of BMI categories by sex. Correlation analysis and scatter plots were used to assess the correlation between BMI and BMC (kg) or w-BMC%. Simple and age-adjusted linear regression models were applied to study the direction and size of the association between w-BMC% and body composition. For this purpose, two models were employed to regress w-BMC% on BMI across three categories and w-BMC% on the trunk fat/ALM ratio while adjusting for age in both males and females. All tests were considered significant at *p* < 0.05. All statistical analyses were conducted with Statistical Package for the Social Sciences (SPSS) version 26 (2019) [31].

## 3. Results

A total of 1404 older adults with a mean age of 67.64 ± 6.32 years and a BMI of 29.09 ± 4.71 kg/m^2^ were included in the study. Males comprised 43.3% (*n* = 608) and females 56.7% (*n* = 796). The mean age did not differ between both genders (67.77 ± 6.30 years vs. 67.53 ± 6.33 years), while males were heavier (83.28 ± 14.22 kg vs. 71.25 ± 12.98 kg) and taller (1.69 ± 0.07 m vs. 1.56 ± 0.07 m) compared to females (*p* < 0.0001). Body composition differed significantly between them, with females having significantly higher BF (31.40 ± 9.53 kg vs. 27.89 ± 9.05 kg) and BF% (43.25 ± 6.84% vs. 32.77 ± 6.78%) (*p* < 0.0001). Trunk fat in females was lower (16.87 ± 5.60 kg vs. 17.49 ± 5.86 kg) (*p* = 0.048) in comparison to males. Trunk fat% was significantly higher in males (62.42 ± 5.39%) relative to females (53.45 ± 6.30%) (*p* < 0.0001). However, females had lower LM (37.79 ± 5.19 kg vs. 52.56 ± 7.12 kg) and LM% (53.82 ± 6.57% vs. 63.77 ± 6.46%) as well as ALM (16.20 ± 2.64 kg vs. 23.12 ± 3.86 kg) than males (*p* < 0.0001). In terms of the trunk fat/ALM ratio, females had significantly higher ratios (1.05 ± 0.31 vs. 0.76 ± 0.24) than males (*p* < 0.0001). BMC (2.05 ± 0.33 kg vs. 2.83 ± 0.45 kg), and w-BMC% (2.93 ± 0.50% vs. 3.46 ± 0.57%) was significantly lower in females than males (*p* < 0.0001) (Table 1).

A total of 608 (43.3%) older adult males with a mean age of 67.77 ± 6.30 years and a BMI of 28.96 ± 4.29 kg/m^2^ were included in this study. The mean age differed significantly across the BMI categories among older male adults, with normal-weight males being older (69.74 ± 6.99 years) compared to those who were overweight (67.51 ± 6.27 years) or with obesity (67.11 ± 5.79 years). Body composition varied considerably across the BMI categories, with those with obesity having the highest total BF (35.76 ± 6.28 kg), followed by overweight (25.45 ± 4.98 kg) and then normal-weight (16.81 ± 5.47 kg) (*p* < 0.0001) individuals. The same trend occurred for BF% (37.27 ± 4.17% vs. 32.05 ± 4.98% vs. 24.99 ± 6.89%) (*p* < 0.0001), trunk fat (22.56 ± 3.89 vs. 15.98 ± 3.28 vs. 10.19 ± 3.72) (*p* < 0.0001), and trunk fat% (63.26 ± 4.12% vs. 62.81 ± 4.79% vs. 59.84 ± 7.71%) (*p* < 0.0001). Alternatively, while LM (46.92 ± 5.04 kg vs. 50.90 ± 5.64 kg vs. 57.00 ± 6.70 kg) and ALM (20.14 ± 3.11 kg vs. 22.48 ± 3.27 kg vs. 25.22 ± 3.58 kg) exhibited the same trend as BF and increased progressively across the BMI categories (*p* < 0.0001), LM% revealed a decreasing trend, with normal-weight older adult males demonstrating the highest LM% (71.06 ± 6.73%), followed by overweight (64.35 ± 4.82%) then obese (59.66 ± 4.07%) (*p* < 0.0001) individuals. When comparing the trunk fat/ALM ratio across BMI categories, the same increasing trend was observed, with older adult males with obesity having a significantly higher ratio (0.91 ± 10.19 vs. 0.73 ± 0.19 vs. 0.52 ± 0.21) (*p* < 0.0001) (Table 2).

**Figure 1:**
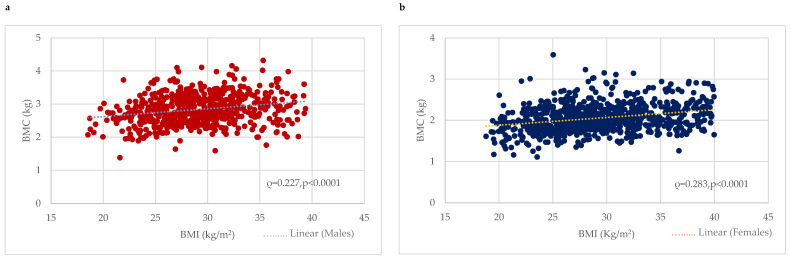
Association between BMI (kg/m^2^) and BMC (kg) among (**a**) males and (**b**) females. BMC = Bone mineral content.

**Figure 2:**
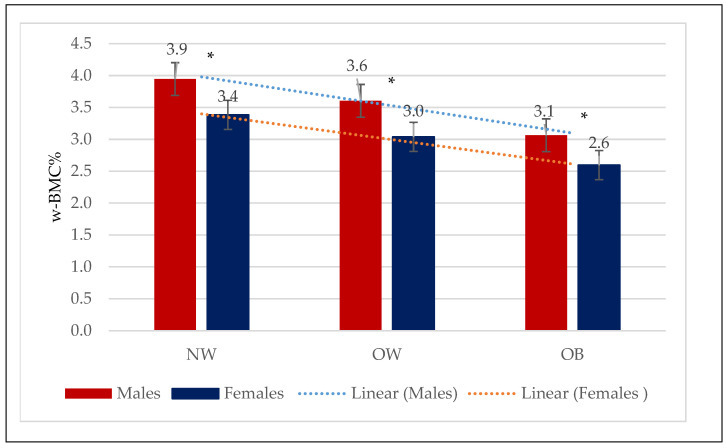
Mean w-BMC% within the three BMI categories by males and females. NW = Normal weight; OW = Overweight; OB = Obesity; w-BMC% = BMC adjusted by body weight expressed as a percentage. * *p*-value for Student’s *t*-test <0.05.

BMC was considerably higher among males with obesity (2.92 ± 0.46 kg) or who were overweight (2.85 ± 0.42 kg) compared to the normal-weight (2.61 ± 0.42 kg) group (*p* < 0.0001). Correlation analysis revealed a significant positive correlation between BMC (kg) and BMI in males (0.227, *p* < 0.0001) (Figure 1). In contrast, w-BMC% exhibited a progressively decreasing trend across the BMI categories, with the highest being in normal-weight individuals (3.95 ± 0.54%), followed by overweight ones (3.60 ± 0.42%), and the lowest among those with obesity (3.06 ± 0.44%) (*p* < 0.0001) (Table 2) (Figure 2).

A total of 796 (56.7%) older females with a mean age of 67.53 ± 6.33 years and BMI of 29.20 ± 5.01 kg/m^2^ were included in this study. Their body composition differed significantly across the BMI categories, with individuals with obesity having the highest total BF (39.89 ± 6.93 kg), followed by overweight (28.70 ± 4.75 kg) and then normal-weight (19.88 ± 4.47 kg) (*p* < 0.0001) ones. The same trend occurred for BF% (47.92 ± 4.36% vs. 42.71 ± 4.90% vs. 35.06 ± 5.97%) (*p* < 0.0001), trunk fat (21.71 ± 4.16 kg vs. 15.43 ± 2.89 kg vs. 10.11 ± 2.93 kg) (*p* < 0.0001), and trunk fat% (54.58 ± 5.72% vs. 53.87 ± 5.82% vs. 50.39 ± 7.25%). Alternatively, while LM (40.90 ± 4.99 kg vs. 36.31 ± 4.26 kg vs. 34.56 ± 3.83 kg) and ALM (17.56 ± 2.60 kg vs. 15.65 ± 2.30 kg vs. 14.58 ± 2.01 kg) exhibited the same trend as body fat and increased progressively across the BMI categories (*p* < 0.0001), LM% revealed a decreasing trend, with the normal-weight older adult females demonstrating the highest LM% (61.55 ± 5.82%), followed by the overweight ones (54.26 ± 4.85%) and then those with obesity (49.48 ± 4.29%) (*p* < 0.0001). When comparing the trunk fat/ALM ratio, an increasing trend was observed, with older adult females with obesity having a significantly higher ratio (0.71 ± 0.24 vs. 1.01 ± 0.23 vs. 1.25 ± 0.25) (*p* < 0.0001) (Table 3).

BMC was significantly higher among females with obesity (2.14 ± 0.33 kg), followed by overweight (2.04 ± 0.3 kg) and normal-weight (1.91 ± 0.33 kg) ones. Correlation analysis revealed a significant positive correlation between BMC (kg) and BMI among females (0.283, *p* < 0.0001) (Figure 1). In contrast, w-BMC% exhibited a progressively decreasing trend across the BMI categories, with the lowest among individuals with obesity (2.60 ± 0.37%), then overweight (3.04 ± 0.37%) ones, and the highest value found among those with a normal weight (3.38 ± 0.48%) (*p* < 0.0001) (Table 3) (Figure 2).

**Figure 3:**
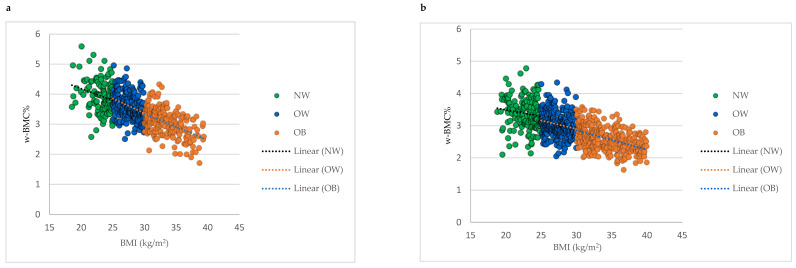
Association between BMI and w-BMC% by BMI category; (**a**) males, (**b**) females. NW = Normal weight; OW = Overweight; OB = Obesity; BMI = Body mass index; w-BMC% = BMC adjusted by body weight expressed as a percentage.

Comparing mean w-BMC% within the BMI categories between male and female older adults revealed a significantly lower w-BMC% among females compared to males within all the BMI categories (Figure 2). The scatter plot in Figure 3 was plotted to examine the variation in w-BMC% with BMI. A clear decreasing trend in w-BMC% was revealed across all the BMI categories with increasing BMI in both male and female older adults. The age-adjusted model among older adults with BMI ≥ 30 kg/m^2^ revealed a significant decreasing trend in w-BMC% among both males (β = −0.09; *p* < 0.0001) and females (β = −0.06; *p* < 0.0001).

**Figure 4:**
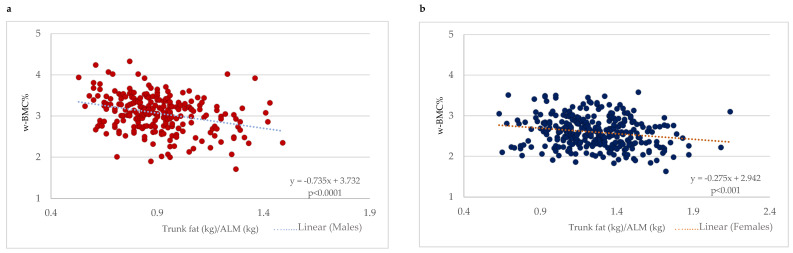
Association between trunk fat/ALM ratio and w-BMC% among participants with BMI ≥ 30 kg/m^2^ stratified by gender; (**a**) males, (**b**) females. ALM = appendicular lean mass; w-BMC% = BMC adjusted by body weight expressed as a percentage.

With a focus on older adults with obesity (BMI ≥ 30 kg/m^2^), a scatter plot between the trunk fat/ALM ratio and w-BMC% was plotted with a simple linear regression model stratified by gender (Figure 4). A significant negative association between the trunk fat/ALM ratio among male and female older adults with BMI ≥ 30 kg/m^2^ was demonstrated (Figure 4). After adjustment for age, the linear regression models still held for a significant decrease of 0.75 percentage points in w-BMC% (β = −0.749; *p* < 0.0001) in males and 0.28 percentage points (β = −0.281; *p* < 0.001) in females for every one-point increase in the trunk fat/ALM ratio.

## 4. Discussion

The main finding of our study is that patients with obesity displayed a lower bone mass in comparison to normal-weight and overweight individuals when taking into account the BMC adjusted by body weight (w-BMC%). We also identified obesity as a negative factor that impacts bone mass, especially in those with central obesity (greater trunk fat deposition) and reduced muscle mass (i.e., appendicular lean mass).

### 4.1. Findings and Concordance with Previous Studies

Our findings are in line with previous works, in which a positive association between BMI and bone mass was found, and obesity was associated with increased BMD [16,22,32] or BMC [28]. In fact, we identified a positive association between BMI and BMC (expressed in kg) in both males and females, and the absolute value of BMC (kg) appeared to be higher in patients with obesity than in overweight individuals and, in turn, those in the normal-weight range. However, some authors consider this positive correlation meaningless, because although a recent study on individuals with obesity (BMI ≥ 30 kg/m^2^) reported higher BMD values in this population [21], this did not imply that they had a lower risk of fracture. In fact, individuals with abdominal obesity (waist obesity > 102 cm), despite their higher BMD, had a greater risk of vertebral fracture when compared to those with a normal weight. The same authors argued that the BMD variable is not the only direct and related factor affecting the fracture risk, as the latter can also be influenced by other aspects [21]. One of these, body weight, is known to impact bone mass [26,27].

In terms of the second finding, when accounting for body weight, a negative association between BMI and weight-adjusted BMC expressed in percentage (w-BMC%) was identified, in which individuals with obesity appeared to display a lower w-BMC% when compared with normal-weight and overweight people. This finding is in line with a recent large-sample Chinese study, in which they found that considering a standardized BMC after adjustment by body weight has led to a decrease in the number of missed diagnoses in patients with a large body weight (i.e., obesity) and reducing misdiagnosis in those with a smaller body weight (i.e., normal-weight individuals) [24]. Therefore, they concluded that BMC adjustment by body weight is always needed to avoid the over- or underestimation of the diagnosis of osteoporosis, and consideration of the BMD or BMC values may lead to misclassification bias [33].

Another factor that may affect bone mass status is body composition, including BF [34]. In our study, while we reported a decrease in w-BMC% across the BMI categories, we also observed increases in BF and BF% across normal-weight to overweight individuals and then individuals with obesity, as expected, and the negative impact of BF on w-BMC% may be due to some hormones secreted by the former. In this regard, advances in research over the last decade have highlighted various hormones involved in the interaction between bone and adipose tissue (i.e., body fat) [35]. For instance, adiponectin is a hormone secreted by adipose tissue that is able to suppress bone resorption through the inhibition of osteoclast differentiation and promotion of osteoblast mineralization activity [35]. However, adiponectin may be negatively associated with body mass (i.e., body weight and BMI) [36], and recent papers have shown that individuals with obesity have lower levels [37,38], which therefore may explain its negative impact on bone in this population. Testosterone is a male sex hormone that acts directly on the osteoblasts by binding to the androgen receptor and consequently promotes bone formation [39,40]. It usually decreases with age to reach low levels in older adults [41], and testosterone levels are also decreased in males with obesity [42]. These two conditions (i.e., age and obesity) may therefore act synergically to accentuate the reduction in testosterone and lessen its protective effect on bones. A third group similar to the previous one, estrogens, which are also sex hormones in females, have a clear beneficial impact on bone metabolism, and their lack leads to age-related bone mass decrease (i.e., osteoporosis) in women after menopause. In postmenopausal women with obesity, the circulating estrogen levels are essentially maintained due to the peripheral aromatization (related to adipose tissue) of increased androgens in relation to insulin resistance. However, the estrogens sourced from adipose tissue have not been found to have a significant protective effect on bones in postmenopausal women [43].

The distribution of body composition may also impact bone mass status, particularly the ratio between lean mass and visceral fat mass [44], and this is in line with our third finding, in which an increased trunk fat/ALM ratio was associated with lower w-BMC%, with the trunk fat representing the central fat deposition. Another important component included in this ratio is the ALM, which expresses the muscle mass of the extremities, whose impact on bone mass is not yet fully understood [45]. Therefore, by including the trunk fat/ALM ratio, we were able to detect the simultaneous impact of central obesity and sarcopenia on bone mass. The underlying mechanism is still unclear, but we speculate that the coexistence of both obesity, especially abdominal obesity [46], and sarcopenia [47] may have a synergistic effect. Chronic inflammation is a common “denominator” seen in both conditions [48] and is known to play a significant role in bone remodeling, specifically in exacerbating it toward a resorption state, leading to a reduction in bone mass [49].

Therefore, the summary of our three findings suggests that obesity seems to interfere with bone metabolism through mechanical (i.e., weight), hormonal (i.e., testosterone, estrogen, adiponectin, and other bone metabolism-related hormones), and inflammatory factors.

### 4.2. Study Strengths and Limitations

Our analysis has certain strengths. To the best of our knowledge, it is one of the few evaluations to investigate bone mass by taking into account body weight status in obesity and comparing it with that in other BMI groups (normal-weight and overweight) in a large sample of older adults of both genders in a “real-world” clinical setting for nutritional management. Secondly, body composition was measured using DXA, which guarantees a precise assessment of the three main body components, namely the bone mineral content, non-bone lean mass, and fat mass, in both the whole body and at the regional level in individuals who are normal-weight, overweight, and with obesity [50,51]. Thirdly, we utilized the BMC rather than the BMD, which showed excellent reproducibility [52] and demonstrated a very good performance in fracture prediction [53], especially when a reduced BMC with normal BMD has been reported [54]. However, our paper also has certain limitations. Firstly, data were collected in a single unit and our results thus require external validation across other populations [55,56]. Secondly, our study is cross-sectional [57], therefore at best it can reveal only an association between obesity and low bone mass, and no cause–effect relationship in which the former influences the latter [58]. Finally, we had no information regarding medications related to bone metabolism, and we also did not perform any objective assessment of lifestyle habits, physical activity levels, or dietary intake, which are factors known to affect body composition and bone mass [59], or biochemical and hormonal blood tests. In regard to the latter, markers [60] such as those related to a person’s chronic inflammatory status may play a central role in the diminished bone mass of individuals with obesity, especially those characterized by increased central fat deposition (abdominal obesity) and decreased lean mass in the extremities (reduced in appendicular muscle mass).

### 4.3. Potential Clinical Implications and New Directions

Our findings have clinical implications in terms of raising awareness among clinicians and patients that obesity is not associated with better bone mass status but instead the opposite. It should therefore not be considered a protective factor and this should be openly discussed with patients. Secondly, these results reveal the importance of regularly assessing bone status in individuals with obesity, especially those with central obesity (i.e., a large waist circumference) and lower muscle mass (i.e., who are at risk of sarcopenic obesity), since this condition seems to be strongly associated with impaired bone status. Moreover, research that can provide a better understanding of the underlying mechanisms behind reduced bone mass in individuals with obesity that can explain the interaction between fat, muscle, and bone under the umbrella of obesity is needed. In addition, new strategies (i.e., nutritional, pharmacological, or involving physical activity, etc.) should be developed that can improve bone mass in order to prevent or reduce the risk of fracture in this specific population.

## 5. Conclusions

Our study found a negative association between body weight status and bone mass, as individuals with obesity appear to display the lowest BMC, especially those with a higher central fat deposition, thus major abdominal obesity, and lower appendicular muscle mass. This represents a new paradigm of the link between obesity and bone status, which contrasts with that of the “obesity paradox” that has been described as a protective general factor in general [19,20] and, as discussed in this paper, specifically with regard to bone status.

## Figures and Tables

**Table 1:** Age, anthropometric measures, and body composition of the study participants by gender (*n* = 1404).

	Total (1404)	Male (*n* = 608)	Female (*n* = 796)	Significance
Age (years)	67.64 (6.32)	67.77 (6.30)	67.53 (6.33)	*p* = 0.477
Weight (kg)	76.46 (14.78)	83.28 (14.22)	71.25 (12.98)	*p* < 0.0001
Height (m)	1.62 (0.09)	1.69 (0.07)	1.56 (0.07)	*p* < 0.0001
BMI (kg/m^2^)	29.09 (4.71)	28.96 (4.29)	29.20 (5.01)	*p* = 0.333
				X^2^ = 0.461; *p* = 0.794
Normal	277 (19.7)	116 (19.1)	161 (20.2)	
Overweight	567 (40.4)	251 (41.3)	316 (39.7)	
Obesity	560 (39.9)	241 (39.6)	319 (40.1)	
BF (kg)	29.88 (9.49)	27.89 (9.05)	31.40 (9.53)	*p* < 0.0001
BF (%)	38.71 (8.56)	32.77 (6.78)	43.25 (6.84)	*p* < 0.0001
Trunk fat (kg)	17.14 (5.73)	17.49(5.86)	16.87 (5.60)	*p* = 0.048
Trunk fat (%)	57.33 (7.40)	62.42 (5.39)	53.45 (6.30)	*p* < 0.0001
LM (kg)	44.19 (9.53)	52.56 (7.12)	37.79 (5.19)	*p* < 0.0001
LM (%)	58.13 (8.18)	63.77 (6.46)	53.82 (6.57)	*p* < 0.0001
ALM (kg)	19.19 (4.71)	23.12 (3.86)	16.20 (2.64)	*p* < 0.0001
Trunk fat/ALM	0.92 (0.32)	0.76 (0.24)	1.05 (0.31)	*p* < 0.0001
BMC (kg)	2.39 (0.55)	2.83 (0.45)	2.05 (0.33)	*p* < 0.0001
w-BMC (%)	3.15 (0.58)	3.46 (0.57)	2.93 (0.50)	*p* < 0.0001

BMI = Body mass index; BF = Body fat; BF (%) = Body fat percentage; LM = Lean mass; LM (%) = Lean mass percentage; ALM = Appendicular lean mass; BMC = Bone mineral content; w-BMC (%) = BMC adjusted by body weight expressed as a percentage.

**Table 2:** Age, anthropometric measures, and body composition among males by BMI category (*n* = 608).

	Total (608)	NW (*n* = 116)	OW (*n* = 251)	OB (*n* = 241)	Significance
Age (years)	67.77 (6.30)	69.74 (6.99) ^a^	67.51 (6.27) ^b^	67.11 (5.79) ^b^	*p* = 0.001
Weight (kg)	83.28 (14.22)	66.35 (7.31) ^a^	79.21 (7.67) ^b^	95.68 (10.68) ^c^	*p* < 0.0001
Height (m)	1.69 (0.07)	1.69 (0.07) ^a^	1.69 (0.07) ^a^	1.70 (0.07) ^a^	*p* = 0.956
BMI (kg/m^2^)	28.96 (4.29)	23.06 (1.58) ^a^	27.57 (1.42) ^b^	33.23 (2.48) ^c^	*p* < 0.0001
BF (kg)	27.89 (9.05)	16.81 (5.47) ^a^	25.45 (4.98) ^b^	35.76 (6.28) ^c^	*p* < 0.0001
BF (%)	32.77 (6.78)	24.99 (6.89) ^a^	32.05 (4.98) ^b^	37.27 (4.17) ^c^	*p* < 0.0001
Trunk fat (kg)	17.49 (5.86)	10.19 (3.72) ^a^	15.98 (3.28) ^b^	22.56 (3.89) ^c^	*p* < 0.0001
Trunk fat (%)	62.42 (5.39)	59.84 (7.71) ^a^	62.81 (4.79) ^b^	63.26 (4.12) ^b^	*p* < 0.0001
LM (kg)	52.56 (7.12)	46.92 (5.04) ^a^	50.90 (5.64) ^b^	57.00 (6.70) ^c^	*p* < 0.0001
LM (%)	63.77 (6.46)	71.06 (6.73) ^a^	64.35 (4.82) ^b^	59.66 (4.07) ^c^	*p* < 0.0001
ALM (kg)	23.12 (3.86)	20.14 (3.11) ^a^	22.48 (3.27) ^b^	25.22 (3.58) ^c^	*p* < 0.0001
Trunk fat/ALM	0.76 (0.24)	0.52 (0.21) ^a^	0.73 (0.19) ^b^	0.91 (0.19) ^c^	*p* < 0.0001
BMC (kg)	2.83 (0.45)	2.61 (0.42) ^a^	2.85 (0.42) ^b^	2.92 (0.46) ^b^	*p* < 0.0001
w-BMC (%)	3.46 (0.57)	3.95 (0.54) ^a^	3.60 (0.42) ^b^	3.06 (0.44) ^c^	*p* < 0.0001

^a, b, c^ Values with different superscripts are significantly different at *p* < 0.05. BMI = Body mass index; BF = Body fat; BF (%) = Body fat percentage; LM = Lean mass; LM (%) = Lean mass percentage; ALM = Appendicular lean mass; BMC = Bone mineral content; w-BMC (%) = BMC adjusted by body weight expressed in percentage.

**Table 3:** Age, anthropometric measures, and body composition among females by BMI category (*n* = 796).

	Total (*n* = 796)	NW (*n* = 161)	OW (*n* = 316)	OB (*n* = 319)	Significance
Age (years)	67.53 (6.33)	67.76 (7.03) ^a^	67.18 (6.20) ^a^	67.78 (6.09) ^a^	*p* = 0.438
Weight (kg)	71.25 (12.98)	56.35 (5.61) ^a^	67.05 (6.43) ^b^	82.93 (9.89) ^c^	*p* < 0.0001
Height (m)	1.56 (0.07)	1.58 (0.07) ^a^	1.57 (0.07) ^b^	1.55 (0.06) ^b^	*p* = 0.001
BMI (kg/m^2^)	29.20 (5.01)	22.69 (1.59) ^a^	27.34 (1.40) ^b^	34.32 (2.90) ^c^	*p* < 0.0001
BF (kg)	31.40 (9.53)	19.88 (4.47) ^a^	28.70 (4.75) ^b^	39.89 (6.93) ^c^	*p* < 0.0001
BF (%)	43.25(6.84)	35.06 (5.97) ^a^	42.71 (4.90) ^b^	47.92 (4.36) ^c^	*p* < 0.0001
Trunk Fat (kg)	16.87 (5.61)	10.11(2.93) ^a^	15.43 (2.89) ^b^	21.71 (4.16) ^c^	*p* < 0.0001
Trunk Fat (%)	53.45 (6.29)	50.39(7.25) ^a^	53.87 (5.82) ^b^	54.58 (5.72) ^b^	*p* < 0.0001
LM (kg)	37.79 (5.19)	34.56(3.83) ^a^	36.31(4.26) ^b^	40.90 (4.99) ^c^	*p* < 0.0001
LM (%)	53.82 (6.57)	61.55(5.82) ^a^	54.26 (4.85) ^b^	49.48 (4.29) ^c^	*p* < 0.0001
ALM (kg)	16.20 (2.64)	14.58 (2.01) ^a^	15.65 (2.30) ^b^	17.56 (2.60) ^c^	*p* < 0.0001
Trunk fat/ALM	1.05 (0.31)	0.71 (0.24) ^a^	1.01 (0.23) ^b^	1.25 (0.25) ^c^	*p* < 0.0001
BMC (kg)	2.05 (0.33)	1.91 (0.33) ^a^	2.04 (0.32) ^b^	2.14 (0.33) ^c^	*p* < 0.0001
w-BMC (%)	2.93 (0.50)	3.38 (0.48) ^a^	3.04 (0.37) ^b^	2.60 (0.37) ^c^	*p* < 0.0001

^a, b, c^ Values with different superscripts are significantly different at *p* < 0.05. NW = Normal weight; OW = Overweight; OB = Obesity; BMI = Body mass index; BF = Body fat; BF (%) = Body fat percentage; LM = Lean mass; LM (%) = Lean mass percentage; ALM = Appendicular lean mass; BMC = Bone mineral content; w-BMC% = BMC adjusted by body weight expressed as a percentage.

## Data Availability

All data generated or analyzed during this study are included in this published article. The dataset in the present study is available upon request.

## References

[1] El Ghoch M., Fakhoury R. (2019). Challenges and new directions in obesity management: Lifestyle modification programs, pharmacotherapy and Bariatric surgery. J Popul. Ther. Clin. Pharmacol..

[2] Blüher M. (2019). Obesity: Global epidemiology and pathogenesis. Nat. Rev. Endocrinol..

[3] Lifshitz F., Lifshitz J.Z. (2014). Globesity: The root causes of the obesity epidemic in the USA and now worldwide. Pediatr. Endocrinol. Rev..

[4] Pi-Sunyer X. (2009). The medical risks of obesity. Postgrad. Med..

[5] Dalle Grave R., Calugi S., El Ghoch M., Marzocchi R., Marchesini G. (2014). Personality Traits in Obesity Associated with Binge Eating and/or Night Eating. Curr. Obes. Rep..

[6] Itani L., Calugi S., Kreidieh D., El Kassas G., El Masri D., Tannir H., Dalle Grave R., Harfoush A., El Ghoch M. (2019). Validation of an Arabic Version of the Obesity-Related Wellbeing (ORWELL 97) Questionnaire in Adults with Obesity. Curr. Diabetes Rev..

[7] Abdelaal M., le Roux C.W., Docherty N.G. (2017). Morbidity and mortality associated with obesity. Ann. Transl. Med..

[8] El Ghoch M., Calugi S., Dalle Grave R. (2018). Weight cycling in adults with severe obesity: A longitudinal study. Nutr. Diet..

[9] Muscogiuri G., El Ghoch M., Colao A., Hassapidou M., Yumuk V., Busetto L. (2021). Obesity Management Task Force (OMTF) of the European Association for the Study of Obesity (EASO). European Guidelines for Obesity Management in Adults with a Very Low-Calorie Ketogenic Diet: A Systematic Review and Meta-Analysis. Obes. Facts.

[10] Durrer Schutz D., Busetto L., Dicker D., Farpour-Lambert N., Pryke R., Toplak H., Widmer D., Yumuk V., Schutz Y. (2019). European Practical and Patient-Centred Guidelines for Adult Obesity Management in Primary Care. Obes. Facts.

[11] Guglielmi V., Bettini S., Sbraccia P., Busetto L., Pellegrini M., Yumuk V., Colao A.M., El Ghoch M., Muscogiuri G. (2023). Beyond Weight Loss: Added Benefits Could Guide the Choice of Anti-Obesity Medications. Curr. Obes. Rep..

[12] Coughlan T., Dockery F. (2014). Osteoporosis and fracture risk in older people. Clin. Med..

[13] Gregson C.L., Armstrong D.J., Bowden J., Cooper C., Edwards J., Gittoes N.J.L., Harvey N., Kanis J., Leyland S., Low R. (2022). Correction: UK clinical guideline for the prevention and treatment of osteoporosis. Arch. Osteoporos..

[14] Zhao L.J., Liu Y.J., Liu P.Y., Hamilton J., Recker R.R., Deng H.W. (2007). Relationship of obesity with osteoporosis. J. Clin. Endocrinol. Metab..

[15] Mendonça F.M., Soares R., Carvalho D., Freitas P. (2022). The impact of obesity on bone health: An overview. Endokrynol. Polska.

[16] Turcotte A.F., O’Connor S., Morin S.N., Gibbs J.C., Willie B.M., Jean S., Gagnon C. (2021). Association between obesity and risk of fracture, bone mineral density and bone quality in adults: A systematic review and meta-analysis. PLoS ONE.

[17] Cao J.J. (2011). Effects of obesity on bone metabolism. J. Orthop. Surg. Res..

[18] Fassio A., Idolazzi L., Rossini M., Gatti D., Adami G., Giollo A., Viapiana O. (2018). The obesity paradox and osteoporosis. Eat. Weight. Disord..

[19] Hainer V., Aldhoon-Hainerová I. (2013). Obesity paradox does exist. Diabetes Care.

[20] Oreopoulos A., Kalantar-Zadeh K., Sharma A.M., Fonarow G.C. (2009). The obesity paradox in the elderly: Potential mechanisms and clinical implications. Clin. Geriatr. Med..

[21] Luo J., Lee R.Y. (2020). How Does Obesity Influence the Risk of Vertebral Fracture? Findings from the UK Biobank Participants. JBMR Plus.

[22] Gonnelli S., Caffarelli C., Nuti R. (2014). Obesity and fracture risk. Clin. Cases Min. Bone Metab..

[23] Piñar-Gutierrez A., García-Fontana C., García-Fontana B., Muñoz-Torres M. (2022). Obesity and Bone Health: A Complex Relationship. Int. J. Mol. Sci..

[24] Liu T.T., Li X.D., Wang W.Z., Zhang J.G., Yang D.Z. (2019). Efficacy of weight adjusted bone mineral content in osteoporosis diagnosis in Chinese female population. Chin. Med. J..

[25] Beck T.J., Petit M.A., Wu G., LeBoff M.S., Cauley J.A., Chen Z. (2009). Does obesity really make the femur stronger? BMD, geometry, and fracture incidence in the women’s health initiative-observational study. J. Bone Min. Res..

[26] Rico H., Revilla M., Villa L.F., del Buergo M.A., Ruiz-Contreras D. (1994). Determinants of total-body and regional bone mineral content and density in postpubertal normal women. Metabolism.

[27] Bedogni G., Mussi C., Malavolti M., Borghi A., Poli M., Battistini N., Salvioli G. (2002). Relationship between body composition and bone mineral content in young and elderly women. Ann. Hum. Biol..

[28] Xu J., Yang D.Z., Ma J.F., Wang W.Z., Zhang J.Y. (2010). Effect of bone mechanical load on peak bone mass and exploration on the way of standardization for bone mass. Mod. Prev. Med..

[29] Weir C.B., Jan A. (2020). BMI Classification Percentile and Cut Off Points. StatPearls.

[30] Faul F., Erdfelder E., Lang A.G., Buchner A. (2007). G*Power 3: A flexible statistical power analysis program for the social, behavioral, and biomedical sciences. Behav. Res. Methods.

[31] IBM Corp (2019). IBM SPSS Statistics for Windows.

[32] Rexhepi S., Bahtiri E., Rexhepi M., Sahatciu-Meka V., Rexhepi B. (2015). Association of Body Weight and Body Mass Index with Bone Mineral Density in Women and Men from Kosovo. Mater. Sociomed..

[33] Zhou L., Peng F.L. (2019). Extension of weight-standardized bone mineral content in osteoporosis diagnosis. Chin. Med. J..

[34] Jain R.K., Vokes T. (2022). Fat Mass Has Negative Effects on Bone, Especially in Men: A Cross-sectional Analysis of NHANES 2011–2018. J.Clin. Endocrinol. Metab..

[35] Niwczyk O., Grymowicz M., Szczęsnowicz A., Hajbos M., Kostrzak A., Budzik M., Maciejewska-Jeske M., Bala G., Smolarczyk R., Męczekalski B. (2023). Bones and Hormones: Interaction between Hormones of the Hypothalamus, Pituitary, Adipose Tissue and Bone. Int. J. Mol. Sci..

[36] Arita Y., Kihara S., Ouchi N., Takahashi M., Maeda K., Miyagawa J., Hotta H., Shimomura I., Nakamura T., Miyaoka K. (1999). Paradoxical decrease of an adipose-specific protein, adiponectin, in obesity. Biochem. Biophys. Res. Commun..

[37] Buechler C., Wanninger J., Neumeier M. (2011). Adiponectin, a key adipokine in obesity related liver diseases. World J. Gastroenterol..

[38] Khoramipour K., Chamari K., Hekmatikar A.A., Ziyaiyan A., Taherkhani S., Elguindy N.M., Bragazzi N.L. (2021). Adiponectin: Structure, Physiological Functions, Role in Diseases, and Effects of Nutrition. Nutrients.

[39] Mohamad N.V., Soelaiman I.N., Chin K.Y. (2016). A concise review of testosterone and bone health. Clin. Interv. Aging..

[40] Shigehara K., Izumi K., Kadono Y., Mizokami A. (2021). Testosterone and Bone Health in Men: A Narrative Review. J. Clin. Med..

[41] Barone B., Napolitano L., Abate M., Cirillo L., Reccia P., Passaro F., Turco C., Morra S., Mastrangelo F., Scarpato A. (2022). The Role of Testosterone in the Elderly: What Do We Know?. Int. J. Mol. Sci..

[42] Fui M.N., Dupuis P., Grossmann M. (2014). Lowered testosterone in male obesity: Mechanisms, morbidity and management. Asian J. Androl..

[43] Corina M., Vulpoi C., Branisteanu D. (2012). Relationship between bone mineral density, weight, and estrogen levels in pre and postmenopausal women. Rev. Med. Chir. Soc. Med. Nat. Lasi..

[44] Li L., Zhong H., Shao Y., Zhou X., Hua Y., Chen M. (2023). Association between lean body mass to visceral fat mass ratio and bone mineral density in United States population: A cross-sectional study. Arch. Public Health.

[45] da Cruz G.F., Lunz T.M., de Jesus T.R., Costa M.B., Vidigal C.V., Albergaria B.H., Marques-Rocha J.L., Guandalini V.R. (2021). Influence of the appendicular skeletal muscle mass index on the bone mineral density of postmenopausal women. BMC Musculoskelet. Disord..

[46] Kolb H. (2022). Obese visceral fat tissue inflammation: From protective to detrimental?. BMC Med..

[47] Bano G., Trevisan C., Carraro S., Solmi M., Luchini C., Stubbs B., Manzato E., Sergi G., Veronese N. (2017). Inflammation and sarcopenia: A systematic review and meta-analysis. Maturitas.

[48] Park C.H., Do J.G., Lee Y.T., Yoon K.J. (2018). Sarcopenic obesity associated with high-sensitivity C-reactive protein in age and sex comparison: A two-center study in South Korea. BMJ Open.

[49] Epsley S., Tadros S., Farid A., Kargilis D., Mehta S., Rajapakse C.S. (2021). The Effect of Inflammation on Bone. Front. Physiol..

[50] Lorente Ramos R.M., Azpeitia Armán J., Arévalo Galeano N., Muñoz Hernández A., García Gómez J.M., Gredilla Molinero J. (2012). Dual energy X-ray absorptimetry: Fundamentals, methodology, and clinical applications. Radiologia.

[51] Micklesfield L.K., Goedecke J.H., Punyanitya M., Wilson K.E., Kelly T.L. (2012). Dual-energy X-ray performs as well as clinical computed tomography for the measurement of visceral fat. Obesity.

[52] De Lorenzo A., Andreoli A., Candeloro N. (1995). Within-subject variability in body composition using dual-energy X-ray absorptiometry. Clin. Physiol..

[53] Curtis E.M., Harvey N.C., D’Angelo S., Cooper C.S., Ward K.A., Taylor P., Pearson G., Cooper C. (2016). Bone mineral content and areal density, but not bone area, predict an incident fracture risk: A comparative study in a UK prospective cohort. Arch. Osteoporos..

[54] Curtis E., Harvey N., D’Angelo S., Cooper C., Taylor P., Pearson G., Cooper C. (2016). Assessment of bone mineral content and fracture risk: A UK prospective cohort study. Lancet.

[55] Patino C.M., Ferreira J.C. (2018). Internal and external validity: Can you apply research study results to your patients?. J. Bras. Pneumol..

[56] Akobeng A.K. (2008). Assessing the validity of clinical trials. J. Pediatr. Gastroenterol. Nutr..

[57] Wang X., Cheng Z. (2020). Cross-Sectional Studies: Strengths, Weaknesses, and Recommendations. Chest.

[58] Lindstrom Johnson S. (2010). Research and statistics: A question of time: Cross-sectional versus longitudinal study designs. Pediatr. Rev..

[59] Rondanelli M., Faliva M.A., Barrile G.C., Cavioni A., Mansueto F., Mazzola G., Oberto L., Patelli Z., Pirola M., Tartara A. (2021). Nutrition, Physical Activity, and Dietary Supplementation to Prevent Bone Mineral Density Loss: A Food Pyramid. Nutrients.

[60] Germolec D.R., Shipkowski K.A., Frawley R.P., Evans E. (2018). Markers of Inflammation. Methods Mol. Biol..

